# A simple and versatile microfluidic device for efficient biomacromolecule crystallization and structural analysis by serial crystallography

**DOI:** 10.1107/S2052252519003622

**Published:** 2019-04-19

**Authors:** Raphaël de Wijn, Oliver Hennig, Jennifer Roche, Sylvain Engilberge, Kevin Rollet, Pablo Fernandez-Millan, Karl Brillet, Heike Betat, Mario Mörl, Alain Roussel, Eric Girard, Christoph Mueller-Dieckmann, Gavin C. Fox, Vincent Olieric, José A. Gavira, Bernard Lorber, Claude Sauter

**Affiliations:** a Architecture et Réactivité de l’ARN, UPR 9002, CNRS, Institut de Biologie Moléculaire et Cellulaire (IBMC), Université de Strasbourg, 15 Rue René Descartes, 67084 Strasbourg, France; b Institute for Biochemistry, Leipzig University, Bruederstrasse 34, 04103 Leipzig, Germany; c Architecture et Fonction des Macromolécules Biologiques, UMR 7257 CNRS–Aix Marseille University, 163 Avenue de Luminy, 13288 Marseille, France; d Université Grenoble Alpes, CEA, CNRS, IBS, 38000 Grenoble, France; e Structural Biology, European Synchrotron Radiation Facility, 38043 Grenoble, France; f PROXIMA 2A beamline, Synchrotron SOLEIL, L’Orme des Merisiers, Saint-Aubin, 91192 Gif-sur-Yvette, France; g Paul Scherrer Institute, Swiss Light Source, Forschungsstrasse 111, 5232 Villigen PSI, Switzerland; h Laboratorio de Estudios Cristalográficos, IACT, CSIC–Universidad de Granada, Avenida Las Palmeras 4, 18100 Armilla, Granada, Spain

**Keywords:** macromolecule, crystallization, counter-diffusion, microfluidics, seeding, ligand soaking, trace fluorescent labeling, serial crystallography, room temperature, protein structure, ChipX3

## Abstract

Determining optimal conditions for the production of well diffracting crystals is a key step in every biocrystallography project. Here, a microfluidic device is described that enables the production of crystals by counter-diffusion and their direct on-chip analysis by serial crystallography at room temperature. Nine ‘non-model’ and diverse biomacromolecules, including seven soluble proteins, a membrane protein and an RNA duplex, were crystallized and treated on-chip with a variety of standard techniques including micro-seeding, crystal soaking with ligands and crystal detection by fluorescence. Furthermore, the crystal structures of four proteins and an RNA were determined based on serial data collected on four synchrotron beamlines, demonstrating the general applicability of this multipurpose chip concept.

## Abstract 2

An innovative microfluidic design (ChipX3) is described for simple biomacromolecule crystallization by counter-diffusion, allowing semi-automated structural analysis by serial crystallography at room temperature. The functionalities of ChipX3 are demonstrated using case studies leading to high-resolution structures of four proteins and an RNA.

## 1. Introduction   

Crystallography plays a central role in contemporary biology because it enables the visualization of the 3D architecture of biological macromolecules, which provides insights into their cellular functions and partnerships on the atomic scale (Giegé & Sauter, 2010 ▸; Jaskolski *et al.*, 2014 ▸). Over the past two decades, the advent of structural genomics and associated high-throughput (HTP) technologies (Vincentelli *et al.*, 2003 ▸; Pusey *et al.*, 2005 ▸), together with dramatic improvements in experimental setups and the computational environment at synchrotron facilities (Terwilliger *et al.*, 2009 ▸; Owen *et al.*, 2016 ▸), have revolutionized the field and led to a torrent of new crystal structures. This productivity boost is clear from the number of structures deposited in the Protein Data Bank (PDB), which recently exceeded 150 000 entries.

In spite of such advances, the time-consuming and costly mapping of reagents and phase space to identify conditions that yield diffraction-quality crystals from a limited amount of the macromolecule remains a bottleneck in crystallographic studies (McPherson & Gavira, 2014 ▸; Luft *et al.*, 2014 ▸; Giegé, 2017 ▸). This process generally involves a trial-and-error sampling of chemical and physical space by screening hundreds of different cocktails composed of buffers at different pH values, various crystallants (salts, alcohols and polymers) and temperature to find at least one appropriate solvent and the right supersaturation conditions. The miniaturization of crystallization assays in microplates with drop volumes of 0.1–1 µl (typically containing 1–10 µg of the macromolecule) and automation of the screening procedure have made this task considerably more efficient, making it possible to successfully conduct a project with only a few milligrams of pure sample (Sauter *et al.*, 2012 ▸).

With the introduction of the first microfluidic systems dedicated to HTP screening 15 years ago, the sample volume required for a single experiment was reduced by another order of magnitude, down to a few nanolitres (Hansen *et al.*, 2002 ▸; Zheng *et al.*, 2003 ▸). Indeed, microfluidics was immediately regarded as a major breakthrough, especially for biochemists dealing with samples that are difficult to purify in large quantities, such as macromolecules from higher eukaryotes, large biological assemblies and membrane proteins (Hansen & Quake, 2003 ▸; van der Woerd *et al.*, 2003 ▸). However, despite their potential, microfluidic technologies have not yet been massively adopted by the global community for crystal growth, as illustrated by the limited number of PDB entries (only about 30 as of March 2019) that specifically cite the use of microfluidic systems. This can be partly explained by the cost of these microsystems and their associated equipment, but also by the difficulty in successfully extracting fragile crystals from the chips or the requirement to reproduce them using conventional crystallization methods before they can be subjected to crystallographic analysis.

To expand the functionality and attractiveness of microchips beyond crystallization and HTP screening, several teams have explored the possibility of analyzing crystals directly in their microfluidic environment (Yadav *et al.*, 2005 ▸; Ng *et al.*, 2008 ▸; Sauter *et al.*, 2007 ▸; Dhouib *et al.*, 2009 ▸; Emamzadah *et al.*, 2009 ▸; Hansen *et al.*, 2006 ▸; Stojanoff *et al.*, 2011 ▸). Various geometries and materials have been tested and have led to promising results in terms of data collection, anomalous phasing or time-resolved applications (Pinker *et al.*, 2013 ▸; Khvostichenko *et al.*, 2014 ▸; Perry *et al.*, 2013 ▸, 2014 ▸). The difficulty of cryopreserving crystals to protect them from radiation damage inside chips, owing to the wide flat surfaces of the device causing vapor condensation and ice formation in the cryojet, was first perceived as an obstacle. However, the recent revival of multi-crystal data-collection techniques at room temperature by the X-ray free-electron laser (XFEL) community has changed the paradigm and popularized serial crystallography (Chapman *et al.*, 2011 ▸; Stellato *et al.*, 2014 ▸; Ayyer *et al.*, 2015 ▸). In this context, microfluidic systems provide promising solutions to prepare, handle and analyze crystals at both synchrotron beamlines and XFELs (Heymann *et al.*, 2014 ▸; Sui *et al.*, 2016 ▸).

In this report, we describe a versatile and low-cost microfluidic chip for crystal production and characterization. This chip was initially designed to miniaturize and facilitate the identification of crystal-growth conditions using the counter-diffusion method and its efficient self-optimizing process (Dhouib *et al.*, 2009 ▸; Pinker *et al.*, 2013 ▸). The latest version of the chip design, called ChipX3, incorporates several improvements in terms of sample injection, reservoir loading and design to allow low-cost manufacturing by injection molding. With ChipX3, we demonstrate that crystals can (i) be easily produced by seeding, (ii) be soaked *in situ* with ligands or (iii) be visualized by fluorescence imaging. In addition, the chip provides a stable platform for crystal storage, handling, shipment and *in situ* analysis by serial crystallography. We illustrate a range of applications for ChipX3 by the crystallization of seven soluble proteins, a membrane protein and an RNA duplex, as well as the structure determination of five ‘non-model’ macromolecules at room temperature using data collected on four beamlines at three different synchrotron sites. This lab-on-a-chip approach simplifies and efficiently miniaturizes the crystallographic structure-determination process, from the sample to its 3D structure, in a single device. It offers a user-friendly, cost-effective solution for routine biocrystallographic investigations at room temperature.

## 2. Materials and methods   

### 2.1. Biomacromolecules, biochemicals and chemicals   

The recombinant proteins used in this work include protease 1 from *Pyrococcus horikoshii* (PhP1), the llama nanobody PorM_02 (Nb02), a lipase from *Thermomyces lanuginosus* (Lip; provided by Macrocrystal Oy, Finland), the CCA-adding enzyme from the psychrophilic bacterium *Planococcus halocryophilus* (CCA), the TonB-dependent heme/hemoglobin outer membrane transporter (OMT) ShuA from the pathogen *Shigella dysenteriae* (OMT ShuA), the human mitochondrial aspartyl-tRNA synthetase (hmDRS) and aspartyl-tRNA synthetase 1 from the bacterium *Thermus thermophilus* (ttDRS), which were purified as described previously (Engilberge *et al.*, 2018 ▸; Duhoo *et al.*, 2017 ▸; Ernst *et al.*, 2018 ▸; Brillet *et al.*, 2009 ▸; Sauter *et al.*, 2015 ▸; Zhu *et al.*, 2001 ▸). Horse hemoglobin was purchased from Sigma. The nine-base-pair RNA duplex [r(CGUGAUCG)dC]_2_ was prepared as described by Masquida *et al.* (1999 ▸). Stock concentrations and storage buffers are indicated in Table 1 ▸.

To facilitate the detection of CCA crystals by trace fluorescent labeling (TFL; Pusey *et al.*, 2015 ▸), the protein was fluorescently labeled with carboxyrhodamine-succinimidyl ester (Invitrogen, catalog No. C6157) as described by de Wijn *et al.* (2018 ▸). The labeled protein solution was stored at 277 K and mixed with the protein stock solution just before preparing crystallization assays as a fraction corresponding to less than 1% of the total protein stock. This solution will be referred to as ‘CCA-TFL’.

The nonhydrolyzable analog of cytidyl triphosphate (CTP) that was soaked into the CCA crystals, cytidine-5′-[(α,β)-methyleno]triphosphate (CMPcPP), was purchased from Jena Bioscience (catalog No. NU-438). The lanthanide complex Tb-Xo4 (commercial name Crystallophore) used to crystallize PhP1 was synthesized as described by Engilberge *et al.* (2017 ▸).

### 2.2. ChipX3 manufacturing   

**Figure 1:**
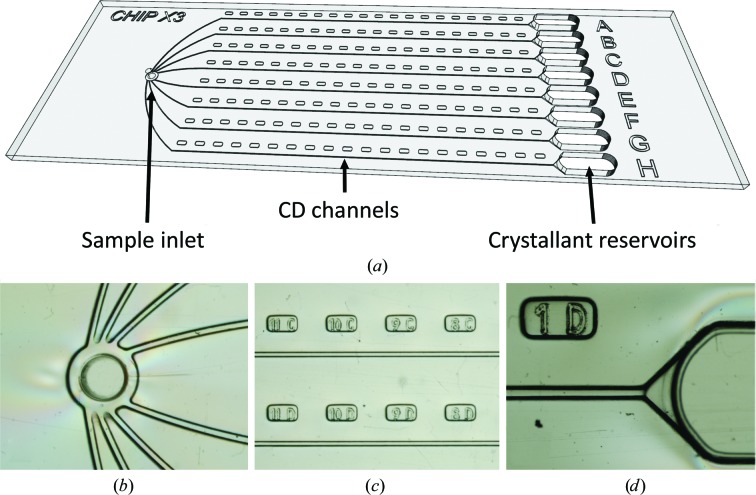
ChipX3 setup. (*a*) Schematic view of the chip, which has the dimensions of a microscope slide (75 × 25 mm) and eight channels with a straight segment of 4 cm and a cross-section of 80 × 80 µm. Close-up views are shown of (*b*) the inlet for the biomacromolecule solution, (*c*) the channels and labels, and (*d*) the end of the channel and the crystallant reservoir.

ChipX3 devices were designed at IBMC, Strasbourg, France in collaboration with Synchrotron SOLEIL, Saint-Aubin, France and were manufactured by MicroLIQUID, Arraste, Spain. The fluidic layer (thickness 1 mm) was produced in cyclic olefin copolymer (COC; TOPAS 5013F-04) by injection molding. Channels and reservoirs were sealed with a second layer of COC (thickness 100 µm). The bonding process was carried out at 398 K and a pressure of 500 kPa. The straight section of the microfluidic channels is 4 cm long with a cross-section of 80 × 80 µm, to give a volume of 260 nl. The reservoir at their extremity has a volume of 10 µl (Fig. 1 ▸).

### 2.3. Sample loading and crystallization   

Crystallization experiments in the ChipX3 were set up in three steps with a conventional 10 µl micropipet (Gilson) and regular tips (StarLab). Firstly, 4–6 µl of macromolecule solution was injected into the sample inlet connecting all channels to fill the entire arborescence up to the reservoirs. Secondly, 1 µl of paraffin oil (Fluka) was injected into the sample inlet to isolate the channels from each other and the inlet was sealed with CrystalClear tape (Hampton Research) to prevent evaporation and solution movements. The third and last step consisted of filling the reservoirs with 5 µl crystallization solution before sealing them with CrystalClear tape. The solutions used to set up the chips are listed in Table 1 ▸. All experiments were incubated at 293 K, except for the RNA duplex, which was crystallized at 310 K.

### 2.4. Crystallization by seeding   

The condition producing the best CCA crystals (de Wijn *et al.*, 2018 ▸) was found using the microseed matrix screening (MMS) method described by D’Arcy *et al.* (2007 ▸, 2014 ▸). Small crystals grown by the hanging-drop method using a reservoir consisting of 1 *M* diammonium hydrogen phosphate, 100 m*M* sodium acetate pH 4.5 (condition E8 from the commercial screen JCSG++ from Jena Biosciences) were recovered, vigorously resuspended, vortexed and diluted in 50 µl of the same crystallant solution. This suspension was stored at 277 K and is referred to as the ‘seed stock’. Protein crystallization solutions were prepared by mixing 6 µl enzyme solution (5.5 mg ml^−1^), 1.5 µl seed stock (either the original or diluted solution) and 1 µl CCA-TFL and were immediately injected into the chip channels. Crystallization of the ttDRS enzyme in the ChipX3 was also performed using seeds. The ‘seed stock’ suspension was prepared as described for the CCA enzyme by crushing crystals grown by vapor diffusion in hanging drops with a reservoir consisting of 7%(*m*/*v*) PEG 8000, 10 m*M* MgCl_2_. ttDRS crystallization solutions were prepared as a mixture consisting of 6.5 µl enzyme solution (19 mg ml^−1^) and 1.5 µl seed stock, either the original or diluted solution, and were immediately injected into the chip channels.

### 2.5. Crystal soaking with substrate   

To soak CCA crystals grown in the ChipX3, the tape covering the reservoirs was removed and 3 µl of 10 m*M* CMPcPP solution was added to selected reservoirs (final concentration of 3.75 m*M*) before sealing them again. This step was performed a week before data collection to ensure good diffusion along the microfluidic channels and in an attempt to maximize site occupancy in the crystals.

### 2.6. X-ray data collection and analysis   

Diffraction data were collected either (i) on beamline PXII (Fuchs *et al.*, 2014 ▸) equipped with a PILATUS 6M detector or beamline PXIII (Bingel-Erlenmeyer *et al.*, 2011 ▸) equipped with a MAR CCD or a PILATUS 2M-F detector at the Swiss Light Source (SLS), Villigen, Switzerland, (ii) on the PROXIMA-2A (PX2A) beamline (Duran *et al.*, 2013 ▸) equipped with an EIGER X 9M detector at SOLEIL, Saint-Aubin, France or (iii) on beamline ID30B (McCarthy *et al.*, 2018 ▸) equipped with a PILATUS3 6M detector at the ESRF, Grenoble, France.

All serial data collections were performed at room temperature (*T* = 293–298 K) on crystals inside ChipX3, owing to the reduced scattering background of the chip (Pinker *et al.*, 2013 ▸). In most cases a dedicated 3D-printed holder mounted on a standard goniometer (see Fig. 5 and Supplementary Fig. S2) was used for data collections. To collect the widest possible rotation angle for each crystal in ChipX3, the channel containing the crystal was aligned with the rotation axis of the goniometer. Crystal alignment was performed either by standard low-dose grid screening at SLS and SOLEIL, or by a one-click procedure at ESRF as described by McCarthy *et al.* (2018 ▸). To avoid collisions with the surrounding equipment (beamstop and collimator), we typically collected 30° rotations per crystal or crystal sector between goniometer positions −30° and +30° (where 0° corresponding to the channels being perpendicular to the X-ray beam). Two data-collection strategies were used to obtain complete data: either merging several partial data sets (sweeps) from the same crystal (one orientation and a wide rotation range) or merging several data sets from different crystals (several orientations and a smaller rotation range per crystal). Table 2 ▸ provides details of data collection and processing.

Partial data sets were individually processed with *XDS* (Kabsch, 2010 ▸). When their number did not exceed ten, they were manually merged with *XSCALE* to find the best combination and determine the appropriate resolution range. In the case of the PhP1 enzyme, *ccCluster* (Santoni *et al.*, 2017 ▸) was used to determine the best partial data sets to merge among the 35 available. For all remaining steps, the *PHENIX* package was used (Adams *et al.*, 2010 ▸). Phases were determined by molecular replacement using the following structures: PDB entries 1miv (Li *et al.*, 2002 ▸) for CCA, 5lmw (Duhoo *et al.*, 2017 ▸) for Nb02, 1g2i (Du *et al.*, 2000 ▸) for PhP1, 4gwl (P. K. Shukla, M. Sinha, J. Mukherjee, M. N. Gupta, P. Kaur, S. Sharma & T. P. Singh, unpublished work) for Lip and 485d (Masquida *et al.*, 1999 ▸) for the RNA. The latter crystals (space group *H*3) presented translational pseudo-symmetry owing to the intrinsic symmetry of the duplex and mero­hedral twinning (twin fraction 0.21–0.39). Hence, the structure was refined using the twin law *h*, −*k* − *h*, −*l*. All structures were built and refined with *Coot* and *phenix.refine* (Emsley & Cowtan, 2004 ▸; Adams *et al.*, 2010 ▸).

## 3. Results and discussion   

### 3.1. ChipX3 design and setup   

ChipX3 was designed to perform counter-diffusion (CD) experiments and take advantage of convection-free conditions (a prerequisite of CD) in channels of small cross-section (width 80 µm, depth 80 µm) to enable the creation of crystallant concentration gradients by pure diffusion [Fig. 1 ▸(*a*)]. The channels, with a length of 4 cm, allow a broad screening of supersaturation states, as does conventional CD in microcapillaries (García-Ruiz *et al.*, 2001 ▸; Otálora *et al.*, 2009 ▸).

The geometry of the sample inlet was adapted to fit standard P2/P10 micropipet tips for chip loading using standard laboratory materials. No extra equipment (such as a pump) is required. The standard micropipet is used to inject the solution into the fluidic system. The branching channel configuration [Fig. 1 ▸(*b*)] allows the simultaneous loading of the eight channels in a single manipulation, thus limiting the loading time and solution dead volumes. Note that in the case of membrane-protein samples containing a detergent (such as ShuA in this work), solutions enter and fill the channels spontaneously owing to capillary action and the native wettability of the COC material. Labels embossed along the channels facilitate crystal location and grid mapping on synchrotron beamlines [Fig. 1 ▸(*c*)].

Once the channels have been filled with the macromolecule solution and the inlet closed with tape, crystallant solutions are deposited in the reservoirs [Fig. 1 ▸(*d*)]. The setup is fully compatible with viscous solutions such as the PEG mixtures used in CD screens (González-Ramírez *et al.*, 2017 ▸). The funnel-like channel shape has been optimized to facilitate the contact between the crystallization and macromolecule solutions and to avoid trapping air bubbles, which could prevent the diffusion process. 1 µl low-gelling temperature agarose solution at 1%(*w*/*v*) can optionally be deposited in the funnel prior to the crystallization cocktail to constitute a physical buffer at the entry to the channels that stabilizes the diffusion interface.

The loading procedure of ChipX3 is fast and straightforward. Setting up a chip with eight different conditions takes less than 5 min even for untrained experimenters, as attested by numerous assays performed in the five laboratories involved in this work and by the many participants of crystallization workshops [FEBS courses in 2014–2018 in Nové Hrady, Czech Republic; International School of Biological Crystallization (ISBC) 2015–2017 in Granada, Spain].

### 3.2. Crystallization in ChipX3   

**Figure 2:**
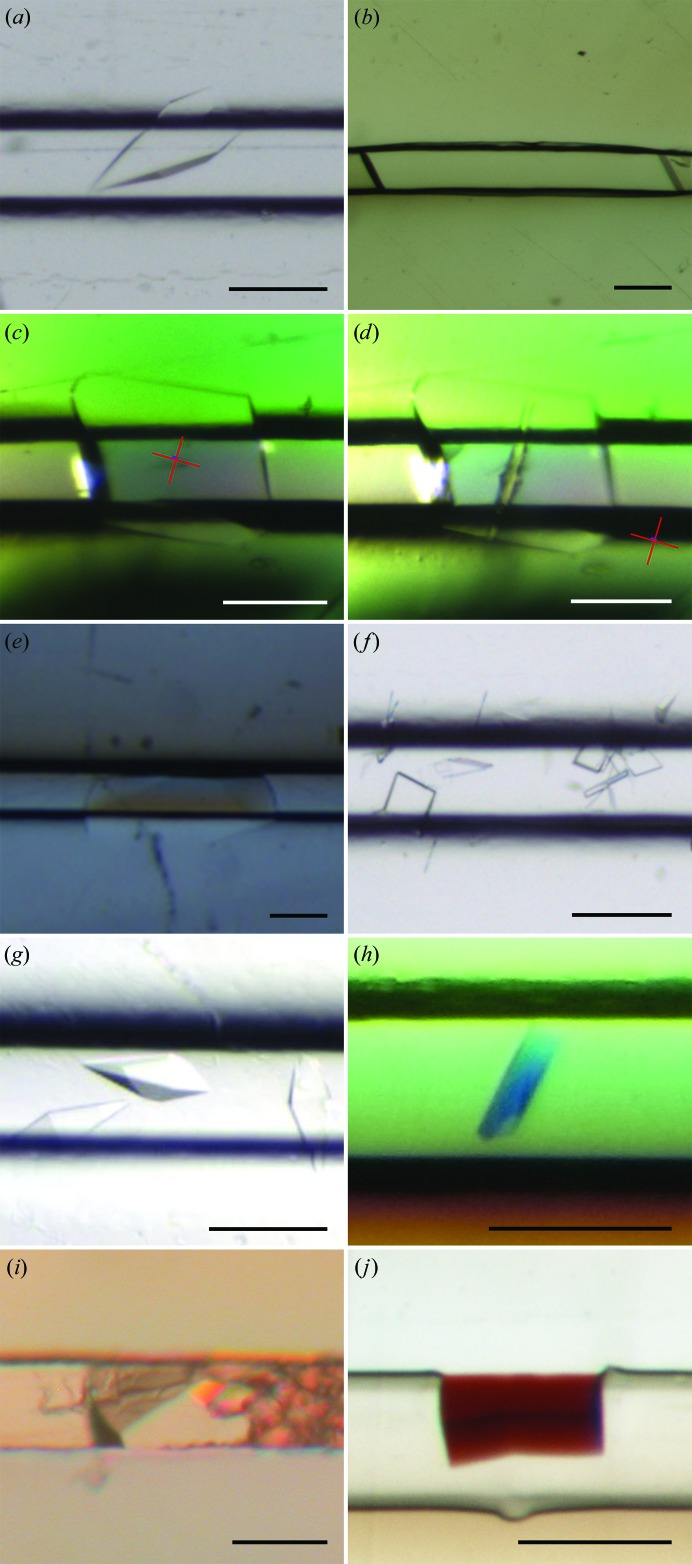
Examples of crystals obtained in ChipX3. Crystals were grown as described in Table 1 ▸. (*a*) CCA, (*b*) PhP1, (*c*, *d*) Nb02 before (*c*) and after (*d*) data collection, with the X-ray beam footprint, (*e*) lipase, (*f*) ttDRS, (*g*) hmDRS, (*h*) OMT ShuA, (*i*) oligo RNA duplex and (*j*) hemoglobin. The scale bar is 0.1 mm in length.

After a prototyping phase of small batches made by hot embossing (Pinker *et al.*, 2013 ▸), a 3D mold was machined with the new ChipX3 specifications to produce a larger batch by injection molding. This enabled validation of the concept using real cases beyond classical model proteins such as lysozyme or thaumatin. We report here on eight proteins of different sizes and sources (from bacteria to human) and an RNA oligomer (Table 1 ▸) crystallized in ChipX3. Crystallization conditions were adapted from those initially used in vapor diffusion or batch crystallization: while the biomacromolecule concentration was kept unchanged, the crystallant concentration was increased by a factor of 1.5–2, as recommended by Otálora *et al.* (2009 ▸). Representative examples ranging from small microcrystals to large crystals filling a portion of the channel are shown in Fig. 2 ▸. Typical counter-diffusion patterns can be observed along the concentration gradient, with microcrystalline material close to the reservoirs where supersaturation is maximal and larger crystals towards the other extremity of the channels (see Supplementary Fig. S1).

**Figure 3:**
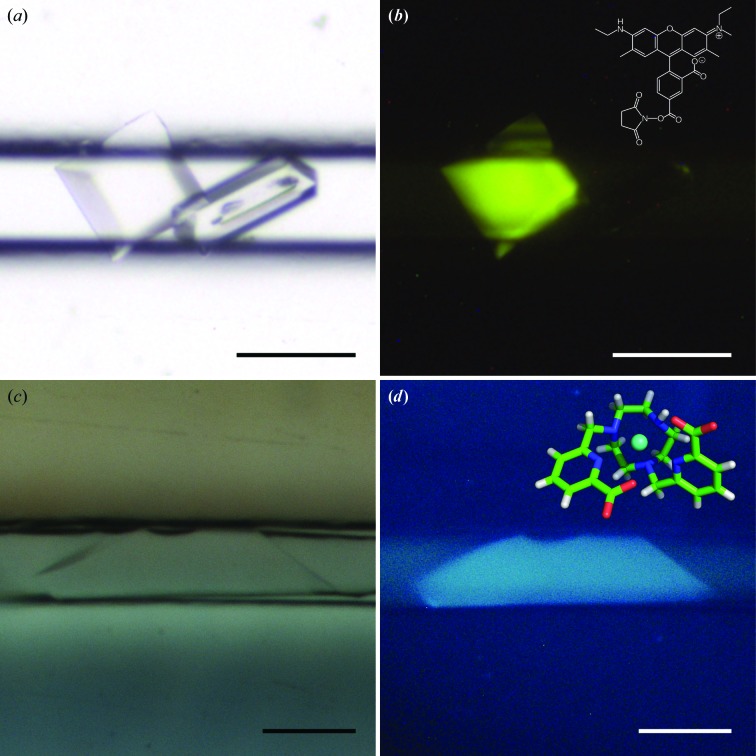
Crystal detection in ChipX3 by fluorescence. (*a*, *b*) CCA crystals grown as described in Table 1 ▸ with 0.6% CCA-TFL; (*c*, *d*) PhP1 crystals grown as described in Table 1 ▸ with 10 m*M* Tb-Xo4. (*a*, *c*) Crystals illuminated with white light. (*b*) Crystal illuminated with a 520 nm light source and image taken with a low-pass filter at 550 nm (LP550); inset, structure of carboxyrhodamine-succinimidyl ester. (*d*) Crystal illuminated with a 280–380 nm UV source; inset, structure of Tb-Xo4. The scale bar is 0.1 mm in length.

Crystals appeared after a few hours or days and could be visualized under polarized light. To facilitate the detection of small crystals, we exploited different fluorescence approaches such as classical UV excitation (Meyer *et al.*, 2015 ▸), the fluorescent lanthanide compound Tb-Xo4 developed by Engilberge *et al.* (2017 ▸) and trace fluorescent labeling (TFL) as developed by Pusey *et al.* (2015 ▸). All three approaches were compatible with ChipX3, but the Tb-Xo4 molecule and TFL gave a much brighter signal (Fig. 3 ▸). Fluorescence has the advantage of rapidly localizing samples in the channels and may be used in the future to automate and speed up serial analysis.

### 3.3. Advanced crystallogenesis strategies   

**Figure 4:**
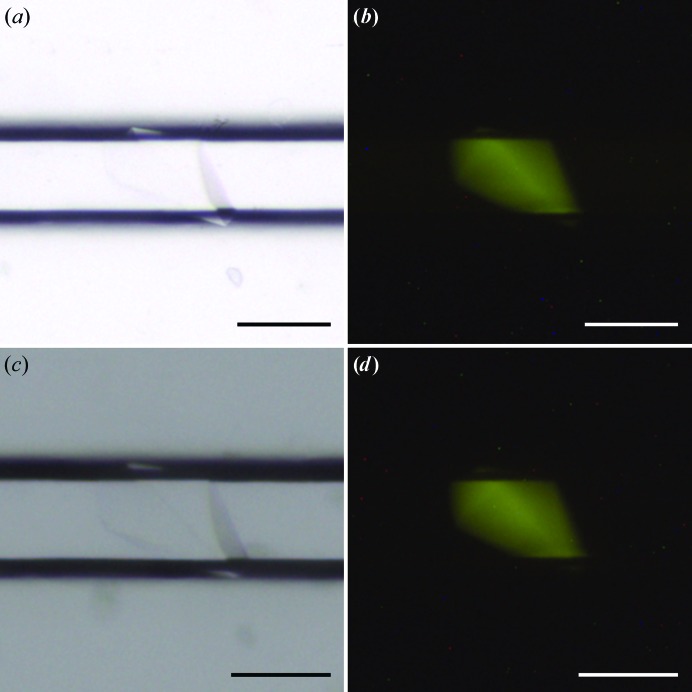
Crystals before and after soaking in ChipX3. Images of CCA crystals grown as described in Table 1 ▸ with 0.6% CCA-TFL. (*a*, *b*) Before soaking. (*c*, *d*) Images taken six days after soaking with CMPcPP at a final concentration of 3.75 m*M*. (*a*, *c*) White-light illumination. (*b*, *d*) Images taken with a 520 nm light source and a low-pass filter at 550 nm (LP550). The scale bar is 0.1 mm in length.

In addition to providing an efficient screening of supersaturation conditions, the CD process has other practical benefits, including the possibility of diffusing anomalous scatterers into pregrown crystals for phasing, or cryoprotecting with compounds such as glycerol (Gavira *et al.*, 2002 ▸; Ng *et al.*, 2003 ▸). In a previous study, we demonstrated the feasibility of on-chip SAD phasing at room temperature using crystals soaked by CD with a lanthanide complex (Pinker *et al.*, 2013 ▸). Along the same lines, CMPcPP, a nonhydrolyzable analog of CTP, which is a substrate of CCA-adding enzymes, was added to the reservoirs once CCA crystals had grown and one week before the synchrotron session. The resulting X-ray structures confirmed that the crystals were derivatized by smooth diffusion without any sign of damage (Figs. 4 ▸ and 6).

Microseeding can also be used together with CD crystallization (Bergfors, 2003 ▸; Gavira *et al.*, 2011 ▸) to bypass the nucleation step and promote rapid crystal growth. Hence, CCA and ttDRS crystals were grown by a combination of CD and seeding. Microseeds were added to the protein solution just before it was injected into the chips and the first crystals appeared in the channels after a few days. Seeding proved to be an effective way to trigger rapid and abundant crystal production, which is of particular interest for serial analysis.

We also used a new nucleant called crystallophore or Xo4 (Engilberge *et al.*, 2018 ▸) in the case of the protein PhP1, for which the crystallization conditions (Table 1 ▸) were determined only in the presence of this terbium complex. Tb-Xo4 was added to the protein solution before filling the channels. It triggered the nucleation and the growth of large PhP1 crystals, which completely filled the available volume. An added value for macromolecules crystallized in the presence of Xo4 is the strong luminescence when illuminated by UV light (see Fig. 3 ▸).

### 3.4. Serial crystal analysis inside ChipX3   

**Figure 5:**
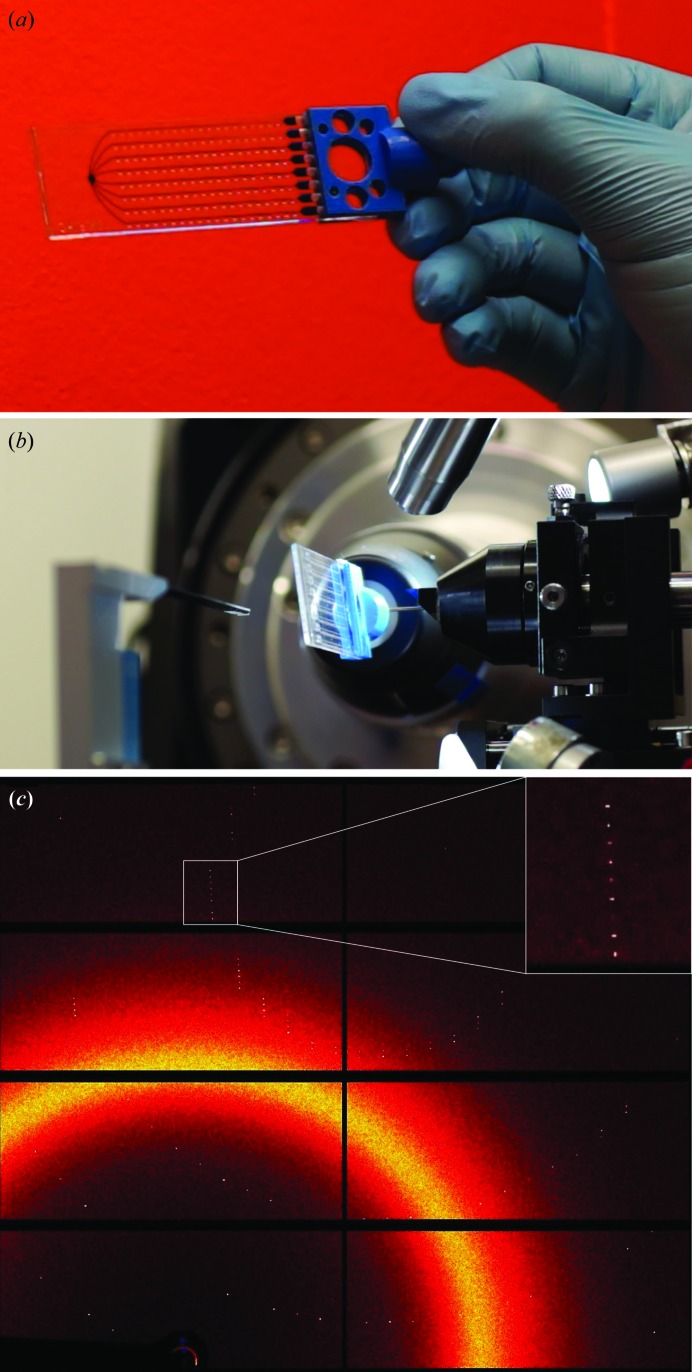
Diffraction analysis in ChipX3. (*a*) ChipX3 on its holder. (*b*) ChipX3 on beamline PXIII at the SLS synchrotron. (*c*) Example of a diffraction pattern of the CCA adding-enzyme in ChipX3 at room temperature (exposure 0.1 s, rotation 0.2°).

The ChipX3 was designed for *in situ* characterization. Its overall thickness was optimized to give a good compromise between material rigidity and X-ray absorption/scattering (Pinker *et al.*, 2013 ▸). The COC material produces a characteristic diffuse scattering ring [Fig. 5 ▸(*c*)] in the resolution range 4–6 Å (see also Fig. 4 in Dhouib *et al.*, 2009 ▸ and Fig. 10.4 in Martiel *et al.*, 2018 ▸), which hardly affects data processing and quality. During data collection, the chip is oriented with its thickest layer facing the direct beam and the thinnest face behind the crystal to minimize the attenuation of the diffraction signal (Fig. 5 ▸). Labels embossed along the channels enable the easy localization of crystals before analysis, with a view to future automation of the procedure on synchrotron beamlines. The chip can also be positioned in the beam using a plate gripper, as illustrated in Supplementary Fig. S3. To avoid intervention from beamline staff to mount/unmount the gripper, we developed a light chip holder that can be directly attached to a standard goniometer. This chip holder is manufactured by 3D printing (Fig. 5 ▸, Supplementary Fig. S2) and integrates a standard metal base (B5, SPINE-style; MiTeGen) that is in contact with the goniometer magnet. The holder can be used with any flat device of microscope-slide dimensions on synchrotron beamlines and laboratory-based instruments. The 3D description file for printing this device is provided as supporting information.

To illustrate the general applicability of on-chip serial crystallography at room temperature, we present the results of structure determination in the 1.5–2.5 Å resolution range of four proteins (CCA, Lip, Nb02 and PhP1) and an RNA (Table 2 ▸, Figs. 5 ▸ and 6 ▸). Hemoglobin crystals also yielded complete data to 2.8 Å resolution (data not shown), whereas the microcrystals of the aspartyl-tRNA synthetases and OMT ShuA only diffracted to low resolution and could not be used for structure determination without further optimization.

The data collections were carried out on series of crystals and their parameters were adapted for crystal size and sensitivity to radiation damage. When collecting several paths from the same crystal signs of radiation damage could clearly be seen [see Fig. 2 ▸(*d*)], accompanied by the formation of gas bubbles as described by Meents *et al.* (2010 ▸) and by the deterioration of data-collection statistics (data not shown).

Note that performing *in situ* analysis, *i.e.* without direct handling of the crystals, is a guarantee that their genuine diffraction properties have been preserved. Comparative tests on thaumatin or lipase crystals in ChipX3 sent by regular postal mail or carried to the synchrotron by experimenters did not show significant differences (results not shown), indicating that the chip is a stable and robust container for crystal storage and transport.

Final crystal structures were obtained either from a single large crystal and two wedges (Nb02) or from combining partial data sets from several individual crystals (RNA, CCA, Lip and PhP1). In the latter case, the use of *ccCluster* considerably facilitated the choice of partial data sets to be merged. The comparison of these structures with equivalent structures solved at cryogenic temperatures only showed small differences (see the r.m.s. distances in Table 2 ▸), although the unit-cell volumes were significantly larger (2.7–6.6%) at room temperature than at 100 K because of crystal shrinkage occurring during cryocooling.

The high sensitivity and low background of the latest hybrid pixel detectors (HPDs) compared with CCD detectors (Pinker *et al.*, 2013 ▸), and the very short analysis time (seconds) of the largest wedge of reciprocal space from single crystals are crucial to outrun radiation damage for room-temperature data collection. The analysis in shutterless mode also limits systematic errors in crystal orientation and thus improves the data quality. For example, the highest apparent mosaicity of the RNA crystals (see Table 2 ▸), which were analyzed at an early stage of this work with a MAR CCD detector, is a direct symptom of the data-collection strategies used before the advent of HPDs. In the future, the widespread integration of HPD technology at synchrotron sites and on laboratory-based X-ray sources will undoubtedly facilitate the development of serial crystallo­graphy.

The concept of serial crystallography was introduced with XFEL sources and their extremely intense X-ray pulses that destroy the sample upon signal emission (a process called ‘diffraction before destruction’). As a consequence, large numbers (thousands) of micro/nanocrystals are necessary to obtain a complete data set from series of individual still images. The serial approach has been extended to room-temperature data collection using synchrotron radiation. However, with a lower beam intensity (compared with XFELs) crystals can be used to collect more than a single image and up to several degrees of rotation. With very stable crystals (see Nb02 in Table 2 ▸), a single crystal may even be sufficient to collect complete data with the help of high symmetry and rapid analysis using HPDs. More generally, the number of crystals that are required for structure determination will depend on their size, their symmetry and their sensitivity to radiation damage. Most of our structures were derived from rather small series of 6–14 crystals and the combination of best data sets (Table 2 ▸). With highly sensitive samples such as membrane proteins, *in situ* room-temperature serial crystallography can still be carried out successfully using several hundred crystals (Huang *et al.*, 2015 ▸). In this context, ChipX3 provides a convenient means to produce batches of crystals distributed along chip channels and, in the future, automatic crystal detection and characterization should contribute to speeding up data collection and popularizing this kind of serial RT analysis.

## 4. Conclusion   

Microfluidics has demonstrated its value in terms of miniaturization for macromolecular crystallization experiments and HTP screening. With ChipX3, we propose a versatile tool that integrates all of the steps of a crystallographic study on a single device with the size of a microscope slide. The same chip serves to produce crystals by counter-diffusion (including seeding techniques), to soak them with ligands (for substrate catalysis, ligand screening in fragment-based drug design or phasing purposes) and to perform their diffraction analysis by *in situ* serial crystallo­graphy. The latter step, which is carried out on-chip at room temperature, no longer requires any crystal handling: neither fishing, nor mounting nor cryocooling. This guarantees the preservation of the intrinsic crystal quality, with the chip being a safe means of sample storage and transportation. ChipX3 is easy to use with standard laboratory equipment for sample loading and crystal observation, making it cost-effective, with minimal training or expertise required. We show the general applicability of this lab-on-chip concept with several case studies. Sample fluorescent labeling, as exemplified in this work, may be exploited to detect and center individual crystals in the X-ray beam and to perform their characterization fully automatically. Such microfluidic devices show great promise in the future in the combination of serial analysis pipelines developed at advanced X-ray sources (XFELs and synchrotrons) for routine structure determination at temperatures close to physiological conditions (Martin-Garcia *et al.*, 2016 ▸; Johansson *et al.*, 2017 ▸).

## Supplementary Material

PDB reference: CCA-adding enzyme, 6ibp

PDB reference: CCA-adding enzyme + CMPcPP, 6q52

PDB reference: nanobody 02, 6gzp

PDB reference: protease 1, 6q3t

PDB reference: lipase, 6hw1

PDB reference: RNA duplex, 6ibq

Supplementary Figures. DOI: 10.1107/S2052252519003622/lz5024sup1.pdf

3D description of the chip holder to be used for 3D printing. DOI: 10.1107/S2052252519003622/lz5024sup2.txt

## Figures and Tables

**Figure 6:**
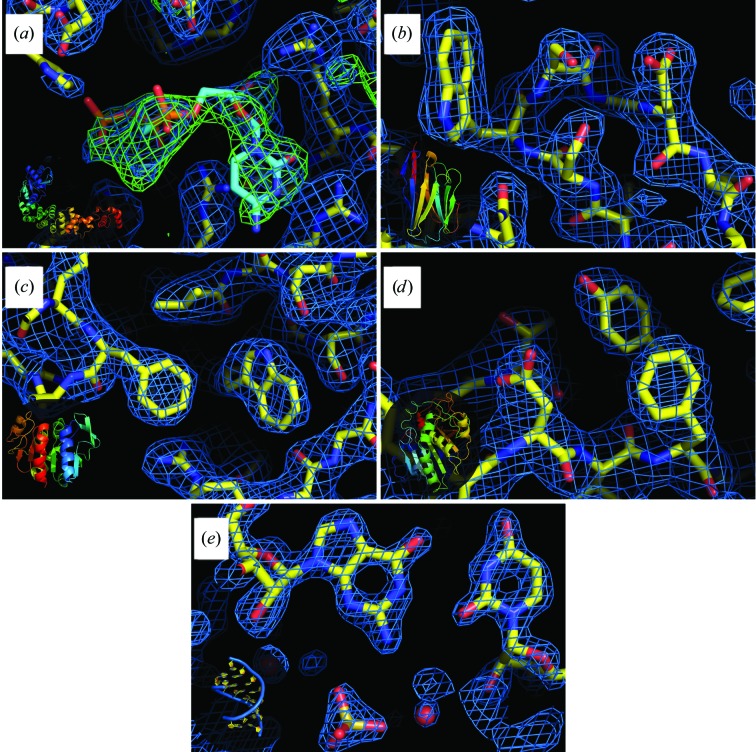
Electron-density maps and structures of target macromolecules. (*a*) CCA-adding enzyme with the positive density from the ligand, (*b*) nanobody, (*c*) protease 1, (*d*) lipase, (*e*) RNA duplex. Insets: schematic representations of the whole macromolecules. This figure was prepared using *PyMOL* (v1.8.6; Schrödinger) with 2*F*_o_ − *F*_c_ electron-density maps (in blue) contoured at 1.2σ and the difference map (in green) contoured at 4σ.

**Table 1:** Biomolecules and crystallization conditions

	Biological source	No. of residues/molecular mass (kDa)	Biomolecule concentration (mg ml^−1^)	Biomolecule buffer solution	Crystallant solution
CCA-adding enzyme	*Planococcus halocryophilus*	420/48.5	5.5	50 m*M* Tris–HCl pH 7.5, 200 m*M* NaCl, 5 m*M* MgCl_2_	30%(*m*/*v*) PEG 3350, 200 m*M* sodium formate pH 6.6
Nanobody 02	Llama	129/14.5	13.8	10 m*M* HEPES–NaOH pH 7.25, 150 m*M* NaCl	20%(*m*/*v*) PEG 3000, 0.1 *M* trisodium citrate pH 5.5
Protease 1	*Pyrococcus horikoshii*	6 × 166/111.6	7.4	20 m*M* Tris–HCl pH 7.5, 10 m*M* Xo4	3.4 *M* malonate pH 7.5
Lipase	*Thermomyces lanuginosus*	269/29.3	30	25 m*M* HEPES–NaOH pH 7.5	0.3 *M* sodium/potasssium phosphate, 50 m*M* sodium acetate pH 4.5
Aspartyl-tRNA synthetase 1	*Thermus thermophilus*	2 × 580/132	19	50 m*M* Tris–HCl pH 7.2, 1 m*M* EDTA, 1 m*M* DTT	10%(*m*/*v*) PEG 8000
Mitochondrial aspartyl-tRNA synthetase	*Homo sapiens*	2 × 630/140	30	50 m*M* HEPES–NaOH pH 7.5, 150 m*M* NaCl, 10% glycerol, 1 m*M* DTT	100 m*M* Tris–HCl pH 7.0, 40%(*m*/*v*) PEG 3350, .2 *M* sodium thiocyanate
OMT ShuA	*Shigella dysenteriae*	632/69.5	20	10 m*M* Tris–HCl pH 8.0, 1.4% β-D-octyl-glucoside	0.1 *M* sodium acetate, 20%(*m*/*v*) PEG 400, 15%(*m*/*v*) PEG 4000, 10%(*m*/*v*) PEG 8000 pH 5.0
RNA duplex	Synthetic	2 × 9/5.8	10	10 m*M* sodium cacodylate pH 6.0, 5 m*M* MgCl_2_	2.6 *M* ammonium sulfate, 50 m*M* sodium cacodylate pH 6.0, 5 m*M* MgSO_4_, 1 m*M* spermine
Hemoglobin	*Equus caballus*	574/62	20	50 m*M* potassium phosphate pH 7.5	3.3 *M* ammonium sulfate, 50 m*M* potassium phosphate pH 7.5

**Table 2:** Data-collection and refinement statistics Values in parentheses correspond to the high resolution range.

	CCA-adding enzyme	CCA-adding enzyme + CMPcPP	Nanobody 02	Protease 1	Lipase	RNA duplex
X-ray beamline	PXIII, SLS	PXII, SLS	PX2A, SOLEIL	PXIII, SLS	ID30B, ESRF	PXIII, SLS
Wavelength (Å)	1.000	1.000	0.826	1.240	0.976	0.826
Temperature (K)	293	293	293	293	293	293
Detector	PILATUS 2M-F	PILATUS 6M	EIGER	PILATUS 2M-F	PILATUS3 6M	MAR CCD
Crystal-to-detector distance (mm)	300	400	154	150/200	502	200
Crystals collected	6	14	9	1/11	14	3
Crystals selected	5	5	1	8	2	3
Rotation range per image (°)	0.1	0.2	0.1	0.2	0.1	2–3
No. of images selected	1000	540	500	1300	600	80
Total rotation range (°)	100	108	50	260	60	155
Exposure time per image (s)	0.1	0.1	0.1	0.1	0.02	1–2
Space group	*P*4_3_2_1_2	*P*4_3_2_1_2	*P*4_3_2_1_2	*P*4_1_2_1_2	*P*6_1_	*R*3
*a*, *c* (Å)	71.5, 293.8	71.4, 293.6	66.7, 91.8	125.6, 133.9	142.6, 80.7	40.0, 69.1
Solvent content (%)	68.3	67.8	65.0	74.0	68.6	54.7
Mean mosaicity (°)	0.04	0.04	0.07	0.04	0.03	0.15
Resolution range (Å)	46–2.54 (2.60–2.54)	48–2.30 (2.40–2.30)	50–2.10 (2.18–2.10)	50–2.15 (2.21–2.15)	49.06–2.50 (2.60–2.50)	23–1.55 (1.59–1.55)
Total No. of reflections	176105 (9374)	232642 (32937)	45307 (4574)	1095436 (85346)	102820 (11312)	21681 (605)
No. of unique reflections	23922 (1598)	34862 (4066)	12281 (1196)	57690 (4522)	31982 (3668)	5485 (304)
Completeness (%)	90.6 (84.6)	99.5 (100.0)	97.2 (98.3)	98.5 (99.6)	98.5 (98.9)	91.5 (69.7)
Multiplicity	7.5 (6.0)	6.7 (8.1)	3.7 (3.8)	19.0 (18.9)	3.2 (3.1)	3.9 (2.0)
〈*I*/σ(*I*)〉	8.1 (1.3)	6.9 (0.7)	11.3 (1.8)	12.0 (1.4)	6.3 (0.8)	6.1 (1.8)
*R*_meas_ (%)	18.9 (126.0)	18.0 (231.2)	7.5 (84.7)	17.4 (206.4)	8.6 (86.8)	17.9 (45.6)
CC_1/2_ (%)	98.7 (55.0)	98.7 (46.9)	99.7 (73.5)	99.7 (69.4)	99.4 (49.4)	98.8 (75.5)
*B* factor from Wilson plot (Å^2^)	57.4	60.6	45.2	50.8	63.3	23.6
Reflections in working/test sets	23583/1180	34840/3405	11053/1228	57659/5758	31516/1573	5484/382
Final *R*_work_/*R*_free_ (%)	18.8/21.4	20.0/22.9	16.9/21.1	16.2/18.4	17.2/19.9	19.2/22.3
No. of non-H atoms						
Total	2998	3028	970	4017	4446	390
Protein	2989	2989	947	3921	4404	342
Solvent	9	10	23	96	47	43
Ligand	0	29	0	0	33	0
Ion	0	0	0	0	2	5
R.m.s.d., bonds (Å)	0.009	0.010	0.008	0.012	0.004	0.004
R.m.s.d., angles (°)	1.23	1.22	0.897	1.43	1.08	0.680
Average *B* factors (Å^2^)						
Overall	60.1	62.6	53.8	57.1	83.8	17.8
Biomolecule	60.1	60.1	53.8	57.1	82.9	17.6
Solvent	52.7	55.5	50.9	56.1	62.6	15.1
Ramachandran plot regions (%)						
Most favored	98.1	97.2	95.8	98.4	96.6	
Allowed	1.9	2.8	4.2	1.6	3.2	
PDB code	6ibp	6q52	6gzp	6q3t	6hw1	6ibq
PDB code at 100 K†	6qy6	6qxn	5lmw	6hf6	4zgb	485d
R.m.s. distance (Å^2^)/Δ*V*_c_ (%)†	0.79/5.0	0.83/3.8	0.79/6.6	0.47/4.3	1.0/3.9	0.40/2.7

† Structures solved at room temperature (this work) are compared with equivalent structures determined at cryogenic temperatures (100 K). R.m.s. distances are calculated taking into account all biomolecule atoms and Δ*V*_c_ quantifies the increase in the unit-cell volume (*V*_c_) at room temperature.
