# Supplementary material for: A simple and versatile microfluidic device for efficient biomacromolecule crystallization and structural analysis by serial crystallography
Source: IUCrJ. 2019 Apr 19;6(Pt 3):454–64. doi: 10.1107/S2052252519003622 (PMC6503916; doi:10.1107/S2052252519003622)
Supplement: Supplementary file 1 [file m-06-00454-sup1.pdf]

IUCrJ

Volume 6 (2019)

Supporting information for article:

A simple and versatile microfluidic device for efficient biomacromolecule crystallization and structural analysis by serial crystallography

Raphaël de Wijn, Oliver Hennig, Jennifer Roche, Sylvain Engilberge, Kevin Rollet, Pablo Fernandez-Millan, Karl Brillet, Heike Betat, Mario Mörl, Alain Roussel, Eric Girard, Christoph Mueller-Dieckmann, Gavin C. Fox, Vincent Olieric, José A. Gavira, Bernard Lorber and Claude Sauter

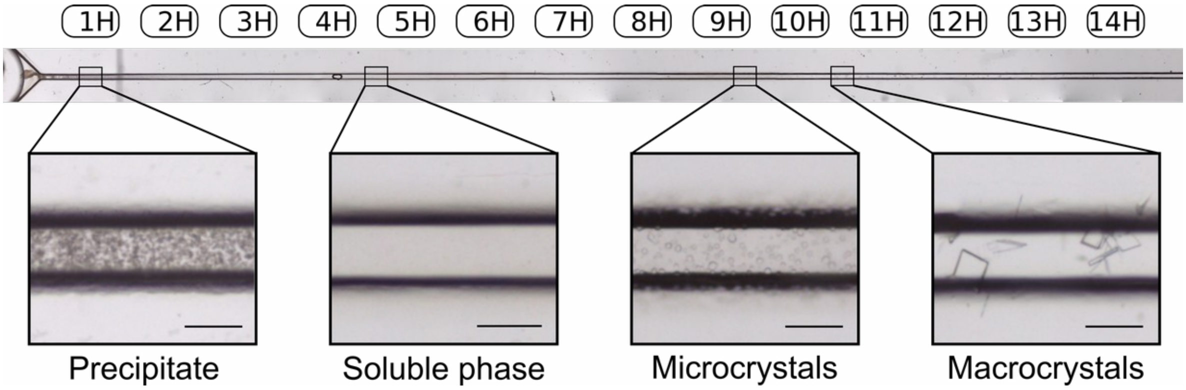

Figure S1: Crystallization of ttDRS by counter-diffusion in ChipX3. Labels printed along the capillaries are represented on top. The displayed linear segment of the channel measures 3 cm. Scale bars = 0.1 mm.

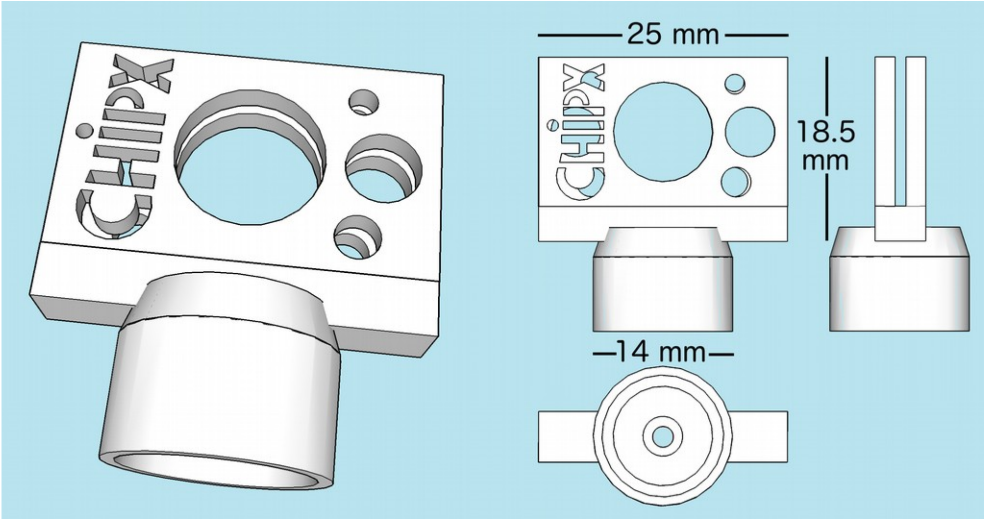

Figure S2: Perspective view and projections of the ChipX3 holder. It was printed using an Ultimaker 2 extended+ printer and a polylactic acid (PLA) filament. After printing, a standard metal base (B5 SPINE style, Mitegen) was glued at the bottom of the holder for coupling with magnetic goniometer heads.

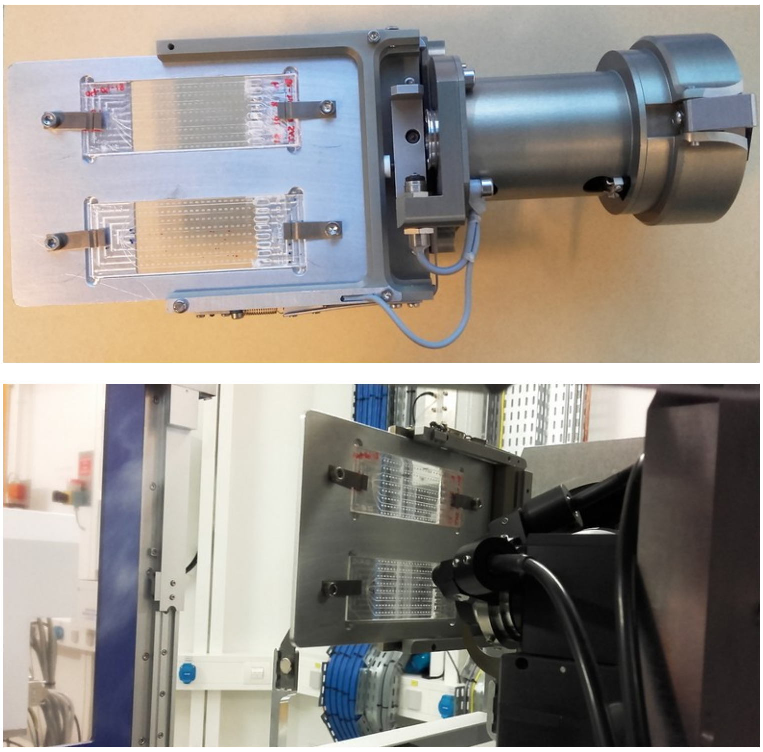

Figure S3: **ChipX3 on ID30B beamline (ESRF, Grenoble)**. (Top) Aluminium holder for two chips, which has the same footprint as a crystallisation microplate and is maintained by a plate gripper designed for *in situ* screening. (Bottom) Plate and gripper mounted on the MD2S diffractometer of ID30B beamline (McCarthy *et al.,* 2018).

Reference:

McCarthy, A. A., Barrett, R., Beteva, A., Caserotto, H., Dobias, F., Felisaz, F., Giraud, T., Guijarro, M., Janocha, R., Khadrouche, A., Lentini, M., Leonard, G. A., Lopez Marrero, M., Malbet-Monaco, S., McSweeney, S., Nurizzo, D., Papp, G., Rossi, C., Sinoir, J., Sorez, C., Surr, J., Svensson, O., Zander, U., Cipriani, F., Theveneau, P. & Mueller-Dieckmann, C. (2018). *Journal of Synchrotron Radiation*. **25**, 1249–1260.
